# Preparation and Characterization of Polybutylene Succinate Reinforced with Pure Cellulose Nanofibril and Lignocellulose Nanofibril Using Two-Step Process

**DOI:** 10.3390/polym13223945

**Published:** 2021-11-15

**Authors:** Azelia Wulan Cindradewi, Rajkumar Bandi, Chan-Woo Park, Ji-Soo Park, Eun-Ah Lee, Jeong-Ki Kim, Gu-Joong Kwon, Song-Yi Han, Seung-Hwan Lee

**Affiliations:** 1 Department of Forest Biomaterials Engineering, Kangwon National University, Chuncheon 24341, Korea; azeliacindradewi@gmail.com (A.W.C.); pojs04@kangwon.ac.kr (J.-S.P.); laa3158@kangwon.ac.kr (E.-A.L.); panda20@kangwon.ac.kr (J.-K.K.); 2 Institute of Forest Science, Kangwon National University, Chuncheon 24341, Korea; rajkumar.pgcb@gmail.com (R.B.); chanwoo8973@kangwon.ac.kr (C.-W.P.); gjkwon@kangwon.ac.kr (G.-J.K.); songyi618@kangwon.ac.kr (S.-Y.H.); 3 National Institute of Forest Science, Seoul 02455, Korea; 4 Kangwon Institute of Inclusive Technology, Kangwon National University, Chuncheon 24341, Korea

**Keywords:** polybutylene succinate, cellulose nanofibril, film composite, twin-screw extrusion

## Abstract

This study reports the preparation of a polybutylene succinate (PBS) film reinforced with pure cellulose nanofibril (PCNF) and lignocellulose nanofibril (LCNF) by a two-step process that consists of solvent dispersion and twin-screw extrusion. Compared to the conventional one-step process, this method offered improved mechanical properties. The addition of 5% CNF increased the tensile properties up to 18.8%. Further, the effect of the lignin content was also studied by using LCNF as a reinforcement. The LCNF was prepared with and without a deep eutectic solvent (DES) pretreatment to gain LCNF with a lignin content that varied between 5, 19, and 30%. The mechanical properties results show that a 5% addition of LCNF to the PBS matrix increased its tensile strength and elastic modulus. Further, the morphological and thermal properties of the composites were also studied in detail.

## 1. Introduction

Polybutylene succinate (PBS) is a biodegradable plastic material that has seen increased interest as an alternative to conventional plastic materials. PBS is a semi-crystalline polymer with high processability and biodegradability, and it has a higher chemical resistance compared to other biodegradable plastics [1,2,3,4]. Because of its properties, PBS has been used in a lot of applications, such as drug capsules, film or rigid packaging, disposable products, and enhancers for other polymers. However, compared to synthetic plastics, PBS application is still limited due to its inadequate mechanical properties. To overcome this problem, reinforcements or fillers are needed to improve the properties of PBS [5].

Cellulose nanofibril (CNF) is a type of nanocellulose with a diameter of 1–100 nm and a micron-scale length [6,7,8]. CNF has high strength properties of around 10 GPa, an elastic modulus in the range of 130 to 140 GPa, a large specific surface area, and high thermal stability [9,10,11,12]. Based on its chemical composition, CNF can be classified into pure cellulose nanofibril (PCNF), lignocellulose nanofibril (LCNF), and holocellulose nanofibril (HCNF) [13]. PCNF contains only cellulose, LCNF contains cellulose, lignin, and hemicellulose, while HCNF contains cellulose and hemicellulose [13,14,15]. CNF can be obtained through the mechanical defibrillation of lignocellulose fibers.

The preparation of CNF through direct mechanical defibrillation is difficult due to the presence of lignin and hemicellulose in the lignocellulose fibers. A pretreatment is used to improve the mechanical defibrillation process. Several pretreatments that can be use are chemical, enzyme, and ionic liquids (IL), including a deep eutectic solvent (DES) pretreatment. Among all these, DES holds great promise for lignocellulose biomass pretreatment due to its good solubility of lignin, low vapor pressure, and viscosity [16,17,18]. Moreover, DES has several advantages, such as low cost, easy preparation, and biodegradable and recyclable properties [19]. DES is prepared by mixing two components: a hydrogen bond donor (HBD) and a hydrogen bond acceptor (HBA). Choline chloride (ChCl) is the most widely used HBA because it is non-toxic, cheap, has renewable merits, and can rapidly form a DES [20]. Meanwhile, commonly used HBDs are lactic acid (LA), formic acid, oxalic acid (OA), and urea [21,22,23]. A DES pretreatment, followed by mechanical defibrillation, offers LCNF. The presence of lignin makes LCNF a suitable material for reinforcing polymers because lignin has hydrophobic properties which are compatible with polymers.

PCNF is a material that is suitable as a reinforcement for biodegradable polymers due to its biodegradability, biocompatibility, and good mechanical properties. Previously, Joy et al. prepared PBS reinforced with isora nanofiber (INF) using a Brabender twin-screw compounder and an injection molding machine [24]. However, the hydrophilic properties of PCNF and the hydrophobic properties of PBS are becoming the main drawbacks that lead to weak interfacial adhesion and bad dispersions of PCNF in the PBS matrix. Mi Zhou et al. reinforced PBS with microfibrillated cellulose (MFC) [25]. To improve the interfacial compatibility, they first treated the MFC by acetylchloride with ball milling. Dispersal in organic solvent and the use of a coupling agent are also good strategies to improve the interfacial adhesion.

In this study, PCNF and LCNF were used as reinforcements for PBS. LCNF with different lignin contents were prepared through DES pretreatment and mechanical defibrillation. Coupling agents were used to improve the interfacial adhesion between PCNF or LCNF and PBS. PBS/PCNF and PBS/LCNF composites were prepared using a two-step process consisting of solvent dispersion and twin-screw extrusion. The effects of the lignin content, coupling agent, and two-step process preparation on the composite properties were investigated.

## 2. Materials and Methods

PBS pellets (Solpol-5000) were purchased from Gio Soltech Co. Ltd. (Wonju, Korea). Commercial PCNF (3.9 wt% in water) was supplied by Cellulose Lab Co., Ltd. (Fredericton, NB, Canada). ChCl, LA, *N*-methyl-2-pyrrolidone (NMP) and Phthalic Anhydride (PA) were purchased from Daejung Chemical & Metals Co., Ltd. (Siheung, Korea). Polymeric methylene diphenyl diisocyanate (PMDI) Cosmonate M-200 was obtained from Kumho Mitsui Chemicals Co. (Seoul, Korea). Korean red pine (*Pinus densiflora* S. et Z.) was obtained from the Experimental Forest of Kangwon National University. The extractives-free wood powder was prepared using an ethanol/benzene (1/2, *v*/*v*) solution in a Soxhlet extractor operating at 90 °C for 6 h.

The PCNF used in this study was prepared from commercial PCNF. Commercial PCNF was diluted to 1.0 wt% in water and pretreated using a high-speed blender at 30,000 rpm for 15 min. The sample was then diluted to 0.1 wt% concentration and subjected to a high-pressure homogenizer (HPH) (MN400BF, Micronox Co., Ltd., Sungnam, Korea). The pressure was set to 20,000 psi, and the defibrillation operation was repeated until the fifth pass.

The ChCl-based DES with LA was synthesized at a molar ratio of 1/1. The mixture of ChCl and LA was stirred at 80 °C until the mixture became a clear liquid. Then, the wood powder (2 g) was added into the DES (98 mL) and stirred at 400 rpm for 24 h at 100 °C, and 130 °C. After the DES treatment, the reactant was centrifuged at 4000× *g* for 20 min, and then the supernatant and slurry were separated. The DES-insoluble residue was washed by vacuum filtration with a 1,4-dioxane/water (4/1) solution followed with water.

The DES-treated product was treated using a high-speed blender at 30,000 rpm for 15 min at a concentration of 1 wt% in water. The sample was then diluted to 0.1 wt% with water and subjected to a high-pressure homogenizer (HPH) (MN400BF, Micronox Co., Ltd., Sungnam, Korea). The pressure was set to 20,000 psi, and the defibrillation operation was repeated until the fifth pass.

LCNF without DES pretreatment was prepared based on the following steps. The extractives-free wood powder was soaked in water (3 wt%). The aqueous suspension was then stirred for 72 h and subjected to a wet disk mill and the mechanical defibrillation was repeated until the tenth pass.

The PBS/PCNF composite, prepared using the one-step method, was directly prepared using a twin-screw extruder. First, the CNF was freeze-dried at −55 °C for 48 h. The freeze-dried CNF and PBS pellets were processed in the twin-screw extruder at 155 °C with a screw rotation speed of 90 rpm, then pelletized and hot-pressed at 120 °C under 10 MPa for 3 min to form uniform films. The PBS/CNF ratio varied between 90/10, 95/5, 97/3, and 99/1.

The PBS/PCNF and PBS LCNF composites were prepared using a two-step process that included solvent dispersion and twin-screw extrusion. In the first step, solvent dispersion, the masterbatch solution was made by putting PBS pellets with or without the coupling agent (PA or PMDI) into 1.5 wt% PCNF or LCNF suspensions in NMP as a solvent. The PBS/PCNF and PBS/LCNF ratio in the masterbatch solution was 90/10. The solution was magnetically stirred at 65 °C for 4 h until the PBS completely dissolved. Then, the solution was poured into a glass petri dish and put in the oven at 65 °C for 24 h for drying. In the second step, the dried PBS/PCNF masterbatch was cut into small pieces and PBS pellets were added to adjust the PBS/PCNF content to 95/5, 97/3, and 99/1, and the PBS/LCNF to 95/5. Table 1 describes the composition for PBS/PCNF. The PBS/PCNF mixture was then processed with a twin-screw extruder at 155 °C with a screw rotation speed of 90 rpm and then pelletized and hot-pressed at 120 °C under 10 MPa for 3 min to form a uniform film. Sample names according to their composition and processing conditions are mentioned in Table 2.

The lignin content in the LCNF products was determined by the Klason method. LCNF (2 g) was added to a 72% sulfuric acid solution (3 mL) and stirred for 2 h at 20 ± 3 °C. Distilled water (112 mL) was subsequently added to the mixture to dilute the sulfuric acid, and the residue was hydrolyzed in an autoclave at 120 °C for 1 h. The acid-insoluble residue was separated by vacuum filtration and washed with excess distilled water until the filtrate was pH-neutral. The lignin content was calculated by comparing the weight of the Klason lignin to the weight of the raw material.

The average diameter of LCNF was calculated based on SEM images. SEM images were captured at a magnification of 50,000. The diameter was measured using ImageJ software. The diameter was taken from at least 500 fibers from SEM images.

The mechanical properties of the neat PBS, as well as the PBS composites, were measured using a universal testing machine (Universal Testing Machine Test One, Model TO-102D, Siheung, Korea) at a cross-head speed of 10 mm/min with a specimen span length of 30 mm. Samples were cut into a dog-bone shape (ASTM D638) and maintained in a thermohygrostat at 25 °C and a relative humidity of 40% to standardize the effect of relative humidity on the tensile properties. At least 5 specimens of each sample were tested, and the average values were taken.

The thermal properties of neat PBS and PBS composites were investigated using a differential scanning calorimeter (SDT Q600, TA Instruments Inc., New Castle, DE, USA) in the Central Laboratory of Kangwon National University. Samples (~5 mg) were heated on a platinum pan in a nitrogen atmosphere. The scanning temperature range was set from −50 to 200 °C, with a heating rate of 10 °C/min. The percentage of crystallinity (%χ_c_) was calculated according to Equation (1):

(1) $$\%\chi_{c}=\frac{\Delta H_{m}}{W_{f}\left(\Delta H_{m}^{0}\right)}$$

where $\Delta H_{m}$ is the melting enthalpy from the DSC data, $\Delta H_{m}^{0}$ is the theoretical melting enthalpy of 100% crystalline polymers (PBS = 110.3 J/g), and $W_{f}$ is the weight fraction of polymers in the composite.

The neat PBS and PBS composite films’ fractured surfaces were examined using a scanning electron microscope (SEM, S-4800, Hitachi, Ltd., Tokyo, Japan) in the Central Laboratory of Kangwon National University. Samples were cryo-fractured using liquid nitrogen and directly used. Prior to observation, the surfaces were sputter-coated with a platinum layer using a high-vacuum sputter coater (EM ACE600, Leica Microsystems Ltd., Wetzlar, Germany) to avoid charging under an electron beam. The morphologies were observed at an accelerating voltage of 5 kV with a working distance of 8.5 mm.

## 3. Results

### 3.1. Lignin Content of LCNFs

The lignin content of LCNF with and without a DES treatment is presented in Table 3. LCNF pretreated with DES at 130 °C has a lignin content of about 5.8 wt% (LCNF5), LCNF pretreated with DES at 100 °C has a lignin content of about 19.8% (LCNF19), and LCNF without DES pretreatment has a lignin content of about 29.9% (LCNF30).

### 3.2. Morphological Characteristics of PCNF, LCNF5, LCNF19, and LCNF30

**Figure 1:**
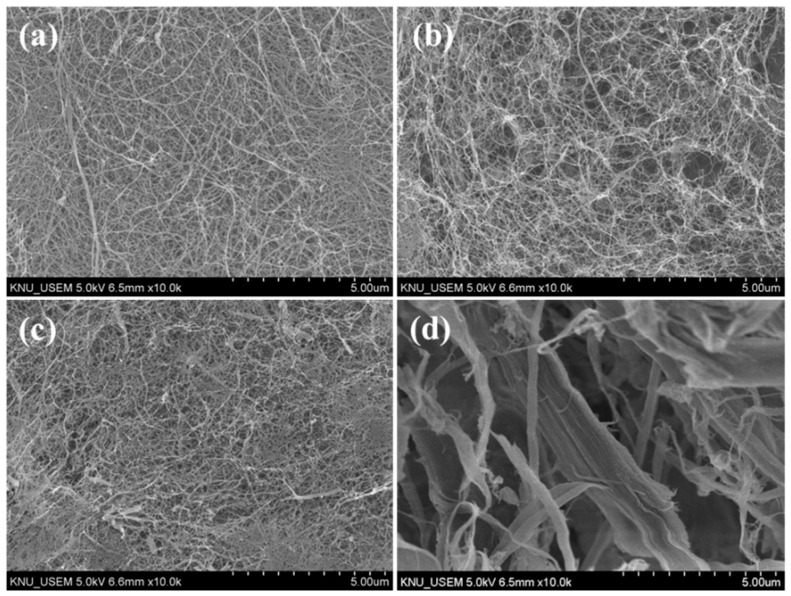
SEM images of PCNF (**a**), LCNF5 (**b**), LCNF19 (**c**), and LCNF30 (**d**).

**Figure 2:**
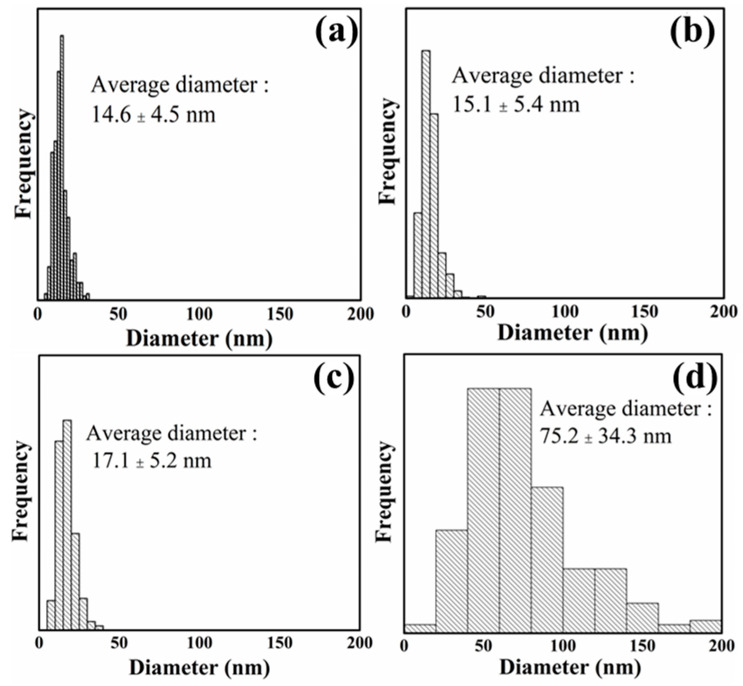
Diameter distribution of PCNF (**a**), LCNF5 (**b**), LCNF19 (**c**), and LCNF30 (**d**).

Morphological characteristics observed by SEM are shown in Figure 1. Average diameters, calculated based on SEM images, are presented in Figure 2. PCNF, LCNF5, and LCNF19 samples exhibited uniform morphologies with interconnected network-like fibrillary structures, whereas the LCNF30 sample presented non-fibrillated thick fibers.

The average diameter of LCNF treated with DES at 130 °C was smaller than that of LCNF treated with DES at 100 °C and LCNF without DES treatment. The average diameter of LCNF decreased with an increase in the DES treatment temperature. As related to lignin content results, samples processed at higher temperatures have lower lignin content; therefore, it improved the defibrillation process. LCNF with DES treatment (LCNF5 & LCNF19) have a diameter around 15–17 nm and the average diameter of LCNF without DES treatment was around 75 nm. This indicates that the DES treatment affects defibrillation efficiency; thus, thinner fibers can be obtained. Removal of lignin leads to the formation of nano space between microfibrils, thus improving the defibrillation efficiency [13].

### 3.3. Morphological Characteristics of PBS/CNF Composites

**Figure 3:**
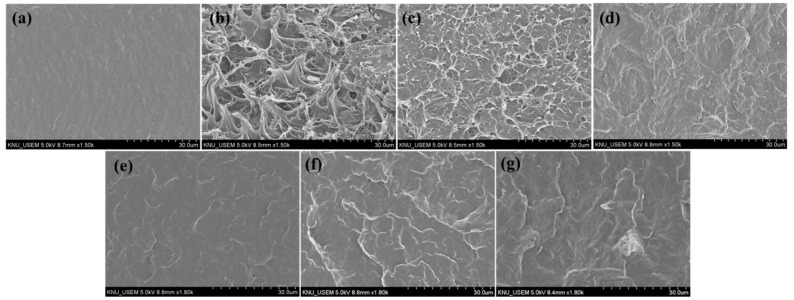
SEM images of the fractured surface of neat PBS (**a**), PBS/PCNF-I (95/5) (**b**), PBS/PCNF-II (95/5) (**c**) PBS/PCNF-II (95/5)PA-1 (**d**), PBS/LCNF5-II (95/5)PA-1 (**e**), PBS/LCNF19-II (95/5)PA-1 (**f**), and PBS/LCNF30-II (95/5)PA-1 (**g**).

A fractured surface morphology of neat PBS, PBS/PCNF, and PBS/LCNF with different lignin contents was observed using SEM analysis. Neat PBS has a smooth fractured surface, as shown in Figure 3a; when 5% of PCNF or LCNF was added, the fractured surface of PBS becomes rough (Figure 3b–g). A rougher surface was observed in the PBS/PCNF composite prepared by the one-step process (Figure 3b). When prepared by the two-step process, the surface becomes smoother (Figure 3c), indicating a better dispersion of CNF in the PBS matrix. With the addition of a 1 phr PA coupling agent to the PBS/CNF composite, an even smoother and homogenous phase appeared. This indicates an improvement in interfacial adhesion when a coupling agent is added. This result was also supported by the mechanical properties results, where a coupling agent further increased the mechanical properties of PBS.

### 3.4. Mechanical Properties of PBS/CNF Composites

**Figure 4:**
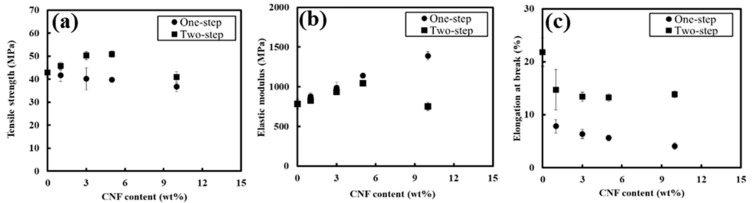
Tensile strength (**a**), elastic modulus (**b**), and elongation at break (**c**) of PBS/PCNF with different PCNF contents and processing.

Mechanical properties were first investigated by comparing PBS/PCNF from the one-step and two-step processes with different PCNF contents. Mechanical property comparisons between PBS/PCNF processed using one-step and two-step processes without a coupling agent are shown in Figure 4. PBS/PCNF processed using the one-step method exhibit a decreasing tensile strength with increasing PCNF content. This is a typical phenomenon in incompatible composites when weak dispersion and interfacial adhesion between the filler and the polymer matrix occurs. The tensile strength of PBS/PCNF prepared using the two-step process was increased from 42.9 MPa of neat PBS to 50.8 MPa of PBS/PCNF with 5% PCNF loading. The elastic modulus was also increased, from 782.7 MPa to 1042.5 MPa. The addition of more than 5% PCNF reduced the flexibility of PBS molecular chains, leading to a decrease in the mechanical properties of the composites [26,27]. Moreover, the two-step process resulted in a higher elongation break compared to the one-step process. In the two-step process, PCNF was, first, solvent-exchanged to NMP, and then PBS was dissolved in the suspensions. This process reduces the hydrophilic properties of PCNF and creates a good dispersion of PCNF in the PBS matrix, thus increasing the mechanical properties [28].

**Figure 5:**
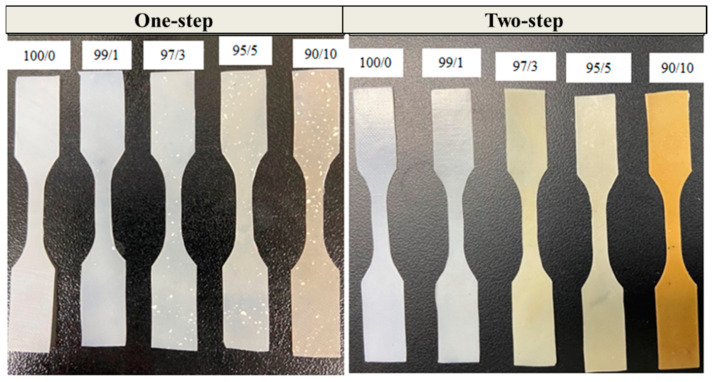
Digital photographs of dog-bone shaped PBS/CNF composites prepared by one-step and two-step processes.

Good dispersions of PCNF in the PBS matrix are also shown from the digital images of PBS/PCNF composite films prepared by the one-step and two-step processes in Figure 5. As can be seen, samples prepared through the two-step process show a uniform dispersion of CNF in the PBS matrix, whereas the samples from the one-step process show a non-uniform dispersion.

**Figure 6:**
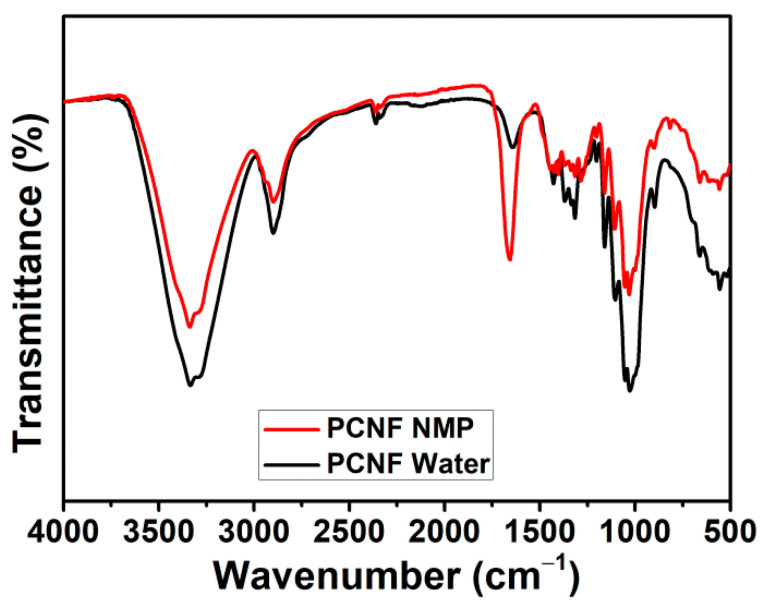
FTIR spectra of PCNF dispersed in water and NMP.

FTIR analysis was performed to gain some insights on the mechanical properties’ improvement. A control PCNF sample (without PBS) was prepared by dispersing it in NMP and oven-drying, according to the procedure described in the experimental section. This is designated as PCNF–NMP. The PCNF–water sample is obtained by directly drying the wet PCNF. The FTIR spectrum of the PCNF–NMP sample is compared with that of the PCNF–water sample to study the influence of NMP dispersion on the hydrophilicity of PCNF. As shown in Figure 6, a clear decrease in the OH peak intensities after NMP treatment, indicating that the PCNF hydrophilicity was decreased. This decreased hydrophilicity will facilitate its better interaction with PBS and will ultimately result in improved mechanical properties.

Based on the mechanical properties results, 5% CNF addition and the two-step process has been chosen for the preparation of PBS/CNF with a coupling agent. The use of a coupling agent is intended to improve the interfacial adhesion between CNF and PBS. PA and PMDI were used as two different coupling agents. The effect of the type and ratio of the coupling agent on mechanical properties is shown in Table 4. The tensile strength of PBS/CNF (95/5), prepared using the two-step process without a coupling agent, was 50.1 MPa; when 1 phr of the coupling agent is added, the tensile strength increased to 57.3 MPa and 54.0 MPa for PA and PMDI, respectively. PA addition has resulted in a slightly higher tensile strength and elastic modulus, compared to PMDI. The addition of 1 phr PA increased the tensile strength by about 30% and the elastic modulus by about 50%. This improvement confirmed that PA and PMDI are improving the interfacial adhesion between the CNF filler and the PBS matrix [27,29]. An overloaded coupling agent is detrimental to the tensile strength of composite [30].

**Figure 7:**
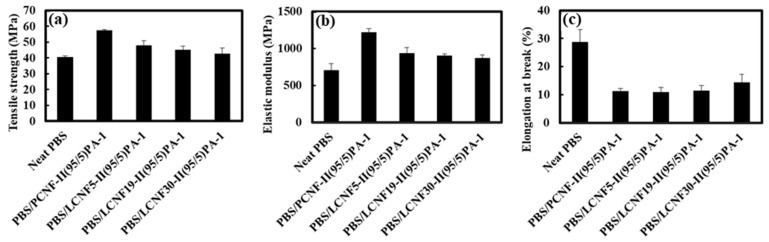
Tensile strength (**a**), elastic modulus (**b**), and elongation at break (**c**) of neat PBS, PBS/PCNF, and PBS/LCNF with different lignin content.

Based on this result, 1 phr PA coupling agent and the two-step process weas used to prepare PBS/LCNF with different lignin contents. Figure 7 shows the mechanical properties result of neat PBS, PBS/PCNF, and PBS/LCNF with different lignin contents. The mechanical properties result shows that a 5% addition of LCNF to the PBS matrix increased the tensile strength and elastic modulus. An increase in tensile properties is related to defibrillation efficiency when lignin content is low (Table 3 and Figure 2). Good mechanical defibrillation decreased the average diameter of LCNF, which in turn increased the specific surface area. This strengthens the hydrogen bonding between nanofibrils and improves tensile strength [31].

### 3.5. Thermal Properties

**Figure 8:**
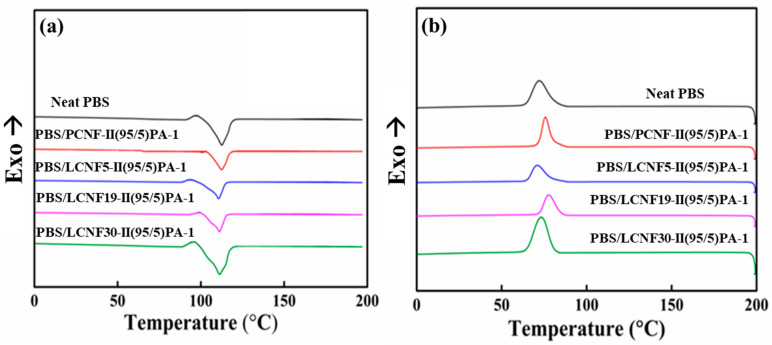
DSC graph of neat PBS, PBS/PCNF, and PBS/LCNF with different lignin content on heating (**a**) and cooling (**b**) cycles.

Thermal properties were analyzed using DSC. A DSC graph of neat PBS and PBS composites is shown in Figure 8. As can be seen in the figure, the addition of either PCNF or LCNF slightly increased the crystallization temperature (T_c_) of PBS. The crystallization temperature (T_c_), melting temperature (T_m_), melting enthalpy (ΔH_m_), and % crystallinity (%χ_c_) of neat PBS, PBS/PCNF, and PBS/LCNF with different lignin contents at a ratio of 95/5 is shown in Table 5.

There were no significant differences in melting temperature (T_m_) between neat PBS and PBS/LCNF composites during the heating stage. The crystallization temperature (T_c_) during the cooling stage of PBS/LCNF5, PBS/LCNF19, and PBS/LCNF30 is 70.64 °C, 77.95 °C, and 73.76 °C, respectively.

### 3.6. Thermal Stability

**Figure 9:**
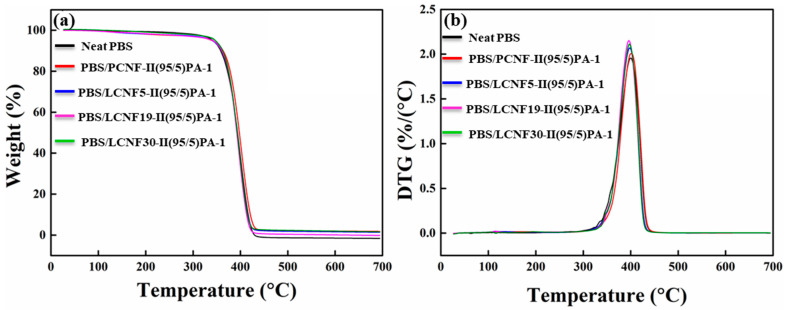
TGA (**a**) and DTG (**b**) graph of neat PBS a, PBS/PCNF (95/5), and PBS/LCNF (95/5) with different lignin contents (Heating rate 10 °C/min).

Thermal stability was investigated using TG analysis. The result of the TG and derivative TG analyses are shown in Figure 9. Thermal degradation of the neat PBS was around 356–427 °C, and when 5% PCNF was added, it was slightly decreased to 338–430 °C. This is probably due to the lower degradation temperature of PCNF. On the other hand, the addition of 5% LCNF slightly increased the thermal degradation temperature to 359–437 °C for PBS/LCNF5, 360–433 °C for PBS/LCNF19, and 361–429 °C for PBS/LCNF30. This can be attributed to the formation of a cross-linked structure due to the presence of lignin, which reduces chain mobility and inhibits chain unzipping during the degradation process [32,33]. The maximum thermal degradation temperature (T_max_) of PBS/LCNF30 was decreased. This is probably due to the presence of hemicellulose which decomposes at low temperatures [34]. As LCNF30 preparation does not involve a DES pretreatment, it is expected that hemicellulose will remain.

## 4. Conclusions

A PBS composite reinforced with PCNF and LCNF with different lignin contents has been successfully prepared using a two-step process. The two-step process involved solvent dispersion and twin-screw extrusion. Compared to one-step twin-screw extrusion process, the mechanical properties of the PBS/CNF composite were improved by the two-step process. It shows that a 5% addition of PCNF or LCNF into the PBS matrix increased the tensile strength and elastic modulus of PBS. Increased mechanical properties can be ascribed to the good dispersion of PCNF and LCNF in the PBS matrix. Furthermore, the interfacial adhesion between both materials was also improved with the addition of 1 phr PA or PMDI coupling agent. This result is supported by a morphological observation of the fractured surface of the composites, which was observed using SEM analysis. SEM images showed no agglomerations of PCNF or LCNF in the PBS matrix. The addition of both PCNF and LCNF also slightly increased the crystallization temperature of PBS, due to the nucleation ability of PBS and LCNF.

## Figures and Tables

**Table 1:** PBS/PCNF composition.

PBS/PCNF Composition (wt%)		PBS/PCNF (90/10) Masterbatch Mixtures (g)	Newly Added PBS (g)
PBS PBS	PCNF PCNF (wt%)		
90	10	20	0
95	5	10	10
97	3	6	14
99	1	2	18

**Table 2:** Sample names in reference to their composition and processing method.

Sample Name	Composition	Processing Method
PBS/PCNF-I(90/10)	PBS to PCNF weight ratio is 90:10	One-step
PBS/PCNF-I(95/5)	PBS to PCNF weight ratio is 95:5	One-step
PBS/PCNF-I(97/3)	PBS to PCNF weight ratio is 97:3	One-step
PBS/PCNF-I(99/1)	PBS to PCNF weight ratio is 99:1	One-step
PBS/PCNF-II(90/10)	PBS to PCNF weight ratio is 90:10	Two-step
PBS/PCNF-II(95/5)	PBS to PCNF weight ratio is 95:5	Two-step
PBS/PCNF-II(97/3)	PBS to PCNF weight ratio is 97:3	Two-step
PBS/PCNF-II(99/1)	PBS to PCNF weight ratio is 99:1	Two-step
PBS/PCNF-II(95/5)PMDI-1	PBS to PCNF weight ratio is 95:5 + 1 phr PMDI coupling agent	Two-step
PBS/PCNF-II(95/5)PMDI-2	PBS to PCNF weight ratio is 95:5 + 2 phr PMDI coupling agent	Two-step
PBS/PCNF-II(95/5)PA-1	PBS to PCNF weight ratio is 95:5 + 1 phr PA coupling agent	Two-step
PBS/PCNF-II(95/5)PA-2	PBS to PCNF weight ratio is 95:5 + 2 phr PA coupling agent	Two-step
PBS/LCNF5-II(95/5)PA-1	PBS to LCNF5 weight ratio is 95:5 + 1 phr PA coupling agent	Two-step
PBS/LCNF19-II(95/5)PA-1	PBS to LCNF19 weight ratio is 95:5 + 1 phr PA coupling agent	Two-step
PBS/LCNF30-II(95/5)PA-1	PBS/LCNF30 is 95:5 + 1 phr PA coupling agent	Two-step

**Table 3:** Lignin content of LCNF.

Sample Code	#DES Pretreatment Temperature (°C)	Lignin Content (wt%)
LCNF5	130	5.8
LCNF19	100	19.8
LCNF30 *	-	29.9

Note: * without DES pretreatment; # Reaction time 24 h.

**Table 4:** Tensile strength, elastic modulus, and elongation at break of neat PBS and PBS/PCNF (95/5) with different coupling agents.

Sample Name	Tensile Strength (MPa)	Elastic Modulus (MPa)	Elongation at Break (%)
Neat PBS	42.90 ± 2.04	782.79 ± 59.72	21.82 ± 2.67
PBS/PCNF-I(95/5)	39.77 ± 0.97	1138.76 ± 16.34	5.58 ± 0.54
PBS/PCNF-II(95/5)	50.81 ± 1.76	1042.85 ± 19.68	13.21 ± 0.68
PBS/PCNF-II(95/5)PA-1	57.33 ± 0.74	1219.70 ± 46.39	11.34 ± 0.83
PBS/PCNF-II(95/5)PA-2	40.66 ± 1.88	785.81 ± 40.02	11.9 ± 1.43
PBS/PCNF-II(95/5)PMDI-1	54.01 ± 1.06	882.42 ± 105.92	19.11 ± 0.68
PBS/PCNF-II(95/5)PMDI-2	45.96 ± 2.14	691.72 ± 35.94	17.22 ± 2.55

**Table 5:** Crystallization temperature (T_c_), melting temperature (T_m_), melting enthalpy (ΔH_m_), and % crystallinity (%χ_c_) of neat PBS, PBS/PCNF, and PBS/LCNF with different lignin content at a ratio of 95/5.

Sample Name	T_c_ (°C)	T_m_ (°C)	ΔH_m_ (J/g)	(%χ_c_)
Neat PBS	70.18	112.43	75.45	68.40
PBS/PCNF-II(95/5)PA-1	76.11	112.77	64.64	63.37
PBS/LCNF5-II(95/5)PA-1	70.64	110.80	69.23	66.07
PBS/LCNF19-II(95/5)PA-1	77.95	111.49	66.50	63.46
PBS/LCNF30-II(95/5)PA-1	73.76	110.87	66.54	63.50

## Data Availability

The data presented in this study are available on request from the corresponding author.
